# Mitigation of PWR fuel assembly vibrations using bio-inspired nozzles

**DOI:** 10.1038/s41598-023-47469-y

**Published:** 2023-11-17

**Authors:** Ibrahim Gad-el-Hak, Njuki Mureithi, Kostas Karazis, Brian Painter

**Affiliations:** 1 Department of Mechanical Engineering, Polytechnique Montréal, Montreal, QC H3C 3A7 Canada; 2 Framatome Inc., 3315 Old Forest Road, Lynchburg, VA 24506 USA

**Keywords:** Mechanical engineering, Marine biology

## Abstract

Jet flows injected in a transverse flow need rapid and effective mixing for various applications ranging from medicine injection into bloodstreams to nuclear pressurized water reactors (PWRs). Inspired by marine organisms, spiral snails, and sharks, bio-inspired nozzles are proposed and experimentally investigated to explore their advantages in suppressing nuclear fuel assembly vibrations. It has been observed that a combination of axial flow and jet cross-flow causes vibrations of fuel rods and potential wear at spacer grid supports. Marine biomimetics is used to improve the mixing between the jet flow and surrounding fluid flows. Inspired by the structure of gastropod shells, a variable whorl spacing nozzle is proposed to induce a swirling jet flow to enhance the mixing rate with the flow inside the reactor cores. In addition, the smooth maneuverability of the sharks highlights the importance to include gill slits structure into nozzles. This work focuses on mitigating PWR fuel assembly vibrations using two biomimetic nozzles, a *snail nozzle* and a *shark nozzle*. These two nozzles are proposed to improve the mixing rate between the injected flow and the primary coolant flow, resulting in a reduced jet flow effect on fuel rods. A single-span mock-up PWR array is designed, fabricated, and instrumented to mimic the real nuclear fuel assembly. The array is experimentally tested under combined axial flow and jet cross-flow to investigate its dynamical behavior. Three different nozzles, a basic circular nozzle, a snail nozzle, and a shark nozzle, are tested. The research investigates the ability of the proposed marine biomimetic nozzles to suppress the vibration of the rod bundle by comparing the results from the three tested nozzles. The obtained results suggest that the proposed snail-inspired biomimetic nozzle is significantly better than the circular nozzle since it reduces rod bundle vibration by increasing flow mixing. A 50% reduction was achieved by implementing it instead of the circular nozzle. More importantly, the shark-inspired nozzle delays the critical jet flow rate, at which the unstable vibration occurs in the rod bundle, by 20%. In addition to delaying instability, a vibration amplitude reduction of 87.5% was obtained using the proposed shark-inspired nozzle compared to the circular nozzle. The results are promising for various applications including gas burners, combustion chambers, and chemical reactors for providing efficient and rapid mixing between two fluid streams.

## Introduction

**Figure 1:**
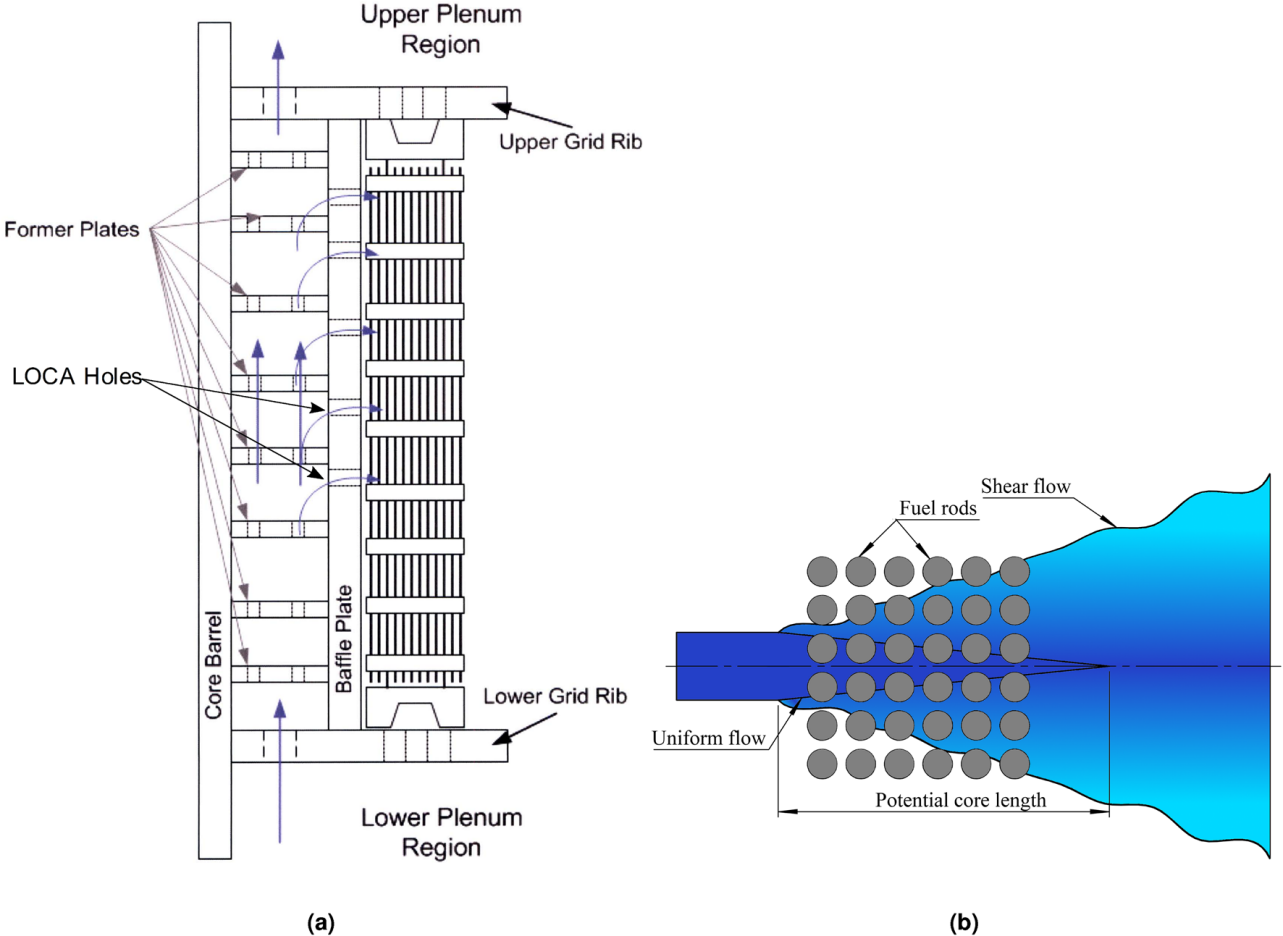
(**a**) Baffle-barrel design including LOCA holes, Ref.^3^ and (**b**) schematic drawing showing flow emanating from LOCA hole towards fuel rods.

Flow-induced vibration (FIV) of fuel rods within fuel assemblies has been the focus of attention in nuclear reactors in order to ensure that nuclear power plant safety is not at risk during normal operation and accident conditions such as a loss-of-coolant accident (LOCA). In pressurized water reactors, fuel assemblies consist of arrays of long small diameter tubes, called fuel cladding, containing fission radioactive products (i.e. fuel rod) and supported at multiple axial locations. In order to remove the heat produced by nuclear fission, fuel rods are subjected to a primarily external axial flow. However, some PWR reactor designs have fail-safe features to release pressure build-up in the event of a loss-of-coolant accident (LOCA) using flow penetrations, LOCA holes, and slots. Thus, at certain locations along the span, a flow component transverse to the rods is also present at the proximity of LOCA holes and slots in the baffles. The safety features including holes and slots in the baffles are shown in Fig. 1. During normal operation, these reactors have experienced grid-to-rod fretting in some of the fuel assemblies due to their proximity to the LOCA holes as reported in a review issued by the International Atomic Energy Agency (IAEA) on fuel failures in water-cooled reactors^1^. The cross-flow components from the LOCA holes and slots are capable of equalizing pressure across the baffles, however, they can induce vibrations higher than those induced by purely axial flows owing to the lower turbulence of external axial flow on the surface of the fuel rods^2^. In extreme cases, the fuel rods may potentially undergo fluidelastic instability which would result in large amplitude vibrations. The dynamic interactions between the fuel rods and their supports “spacer grids” can lead to fuel cladding fretting and the release of radioactive fission products in the primary coolant. Grid-to-rod-fretting (GTRF) failures can thus contribute to increase plant radiation levels during reactor operation. Mitigating fuel rod vibrations is essential to keep fuel cladding robust and intact during its lifetime.

**Figure 2:**
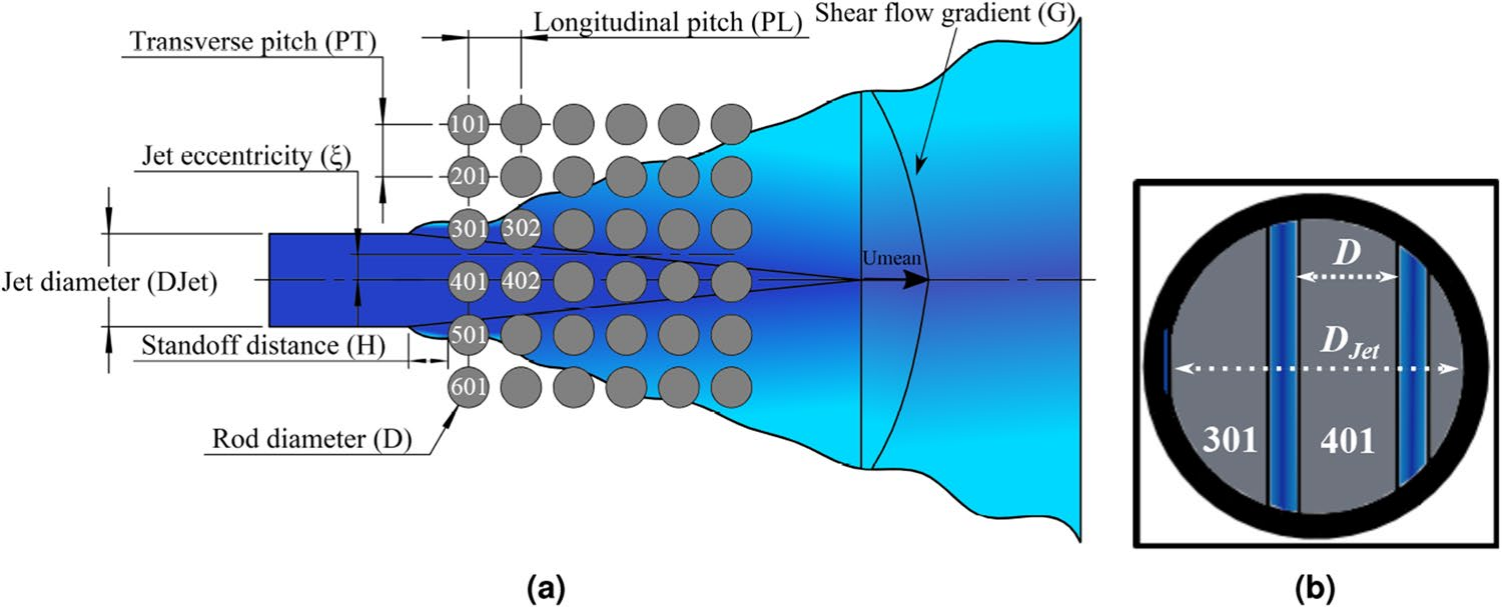
(**a**) Top sectional view of rod array for jet eccentricity of $\xi$=0.5P, and (**b**) projected view from the nozzle at $\xi$=0.25P.

Circular jet cross-flow induced vibrations have been recently investigated to determine the fundamental mechanisms underlying fuel rod excitation. Jet cross-flow, in the absence of axial flow, injecting from LOCA hole has been investigated for prototypical fuel assemblies (i.e. square arrays), as shown in Fig. 1, to study its effect on the fuel rod array stability as reported in Refs.^2,4–10^. The jet cross-flow was found to cause significant amplitude vibrations in the tested arrays. Gad-el-Hak et al.^10^ performed a theoretical study to investigate the fundamental mechanisms underlying fuel rod excitation. The theoretical results confirmed that the jet cross-flow induced fluidelastic instability. The experimental work showed that the critical jet velocity depended on jet flow parameters: (i) jet eccentricity ($\xi$), (ii) jet stand-off distance (*H*), and (iii) jet-to-rod diameter ratio ($D_{Jet}/D$); these parameters are defined in Fig. 2. The instability in the tested array was initiated earlier with an offset of 25% of the pitch. Asymmetrical flow conditions around the rods, which increased the sensitivity of fluidelastic forces due to rod motion, had been created due to this jet eccentricity. To investigate the effect of pure jet cross-flows on stream-wise and transverse vibrations, Gad-el-Hak et al.^6,7^ used asymmetrically flexible rods. The instability in the transverse direction was more significant than that happened in the stream-wise direction. Gad-el-Hak et al.^8,9^ studied the effect of jet-to-rod diameter ratio ($D_{Jet}/D$) on a 6x6 axisymmetric flexible rod bundle. The stability limit increased with decreasing the nozzle diameter. The reduced velocity ($V_{Jet}/fD$) varied linearly with three non-dimensional parameters, the mass damping parameter ($m$ $\delta _{0}$/$\rho$ $D^2$), the reduced jet diameter (*D*/$D_{Jet}$) and the rod span to jet diameter ratio (*L*/$D_{Jet}$). The results suggested that the array lost its stability when the jet momentum exceeded a certain value (i.e. critical jet momentum). On a more practical level, a new bio-inspired nozzle, as demonstrated by Gad-el-Hak and Mureithi^5^, was used to mitigate pure jet cross-flow induced vibrations. The bio-inspired nozzle was experimentally tested versus a circular nozzle on the axisymmetric rod bundle vibration. The comparison revealed that utilizing the biomimetic nozzle delayed the critical jet momentum by 20% and damped the vibration amplitude by 85%.

Current PWR fuel assemblies that have fail-safe features (LOCA holes and slots) suffer from grid-to-rod fretting issues due to combined axial flow and jet cross-flow induced vibrations. In the reactor design, a basic circular shape for LOCA holes has been suggested because it is easier to drill holes in the baffle plates. The ultimate solution to suppress the fuel assembly vibrations from LOCA hole jetting in reactors is improving the mixing rate between jet flow and axial flow which would reduce the penetration depth of jet cross-flow, which would reduce the susceptibility of the fuel rods to transverse flow components. The resulting vibrations would be reduced by converting the jet flow linear momentum to angular momentum (i.e. vortices). In passive jet flow control, the key factor is to change the shape of the circular nozzles in order to improve the mixing rate by inducing swirl flow. Thus, the fuel assembly vibrations due to combined jet cross-flow and axial flow could be mitigated by integrating passive jet control features in LOCA holes. Existing and near-future technologies that rely on standard design and manufacturing techniques are not sufficient to address a suite of urgent engineering problems facing our society, including challenges in nuclear engineering applications. A bio-inspired design concept is utilized to propose two nozzles inspired by the structure of gastropod shells and shark gill slits. As a result, integrating mixing improvement features on a standard LOCA hole shape will be a step in advance over circular nozzles.

The work reported in this paper proposes biomimetic nozzle designs to mitigate the vibration of the fuel assembly due to the combined jet cross-flow and axial flow in pressurized water reactors (PWRs). By using marine biomimetics, we propose new nozzle shapes to be integrated into circular shapes. Inspired by a spiral snail shell structure, we propose a marine biomimetic nozzle to create an intense swirling jet flow. In addition, a second biomimetic nozzle design based on the shark gill slits is tested to determine its effect on the fuel assembly vibrations. A basic circular nozzle is also tested in order to assess the relative ability of the proposed bio-inspired nozzles to reduce vibrations caused by the combined jet cross-flow and axial flow. A reduced array of PWR fuel assemblies is designed, fabricated, and instrumented to measure its vibration under the combined flow. The dynamical behavior of the PWR mock-up bundle is obtained by increasing the jet flow rate through the nozzle up to the occurrence of fluidelastic instability. The array stability thresholds are obtained and collected for each nozzle to compare their abilities to suppress the post-instability vibration amplitudes of the mock-up array.

## Biomimetic design and experimental setup

### Biomimetic design

Passive flow control of jets has been an active subject of research and technology development with the purpose of enhancing the mixing of jet flow with its surrounding domain by a large number of researchers^11–15^. Tab-controlled jets are one of the most effective methods used for increasing entrainment rate of jets by attaching mechanical tabs at the exit plane of nozzles. Tab geometry, tab number, tab orientation, tab size, and tab position relative to the nozzle outlet are the key parameters of tab-controlled jets. These parameters need to be optimized for each application. Recently, a concept of using bio-inspired tabs to improve the mixing effect of circular jet flow has been introduced by Gad-el-Hak and Mureithi^5^. The results of using the bio-inspired nozzle showed a significant impact on the mixing rate of jet flow.

Creating swirl flow is an important factor in jet flow mixing dynamics. In general, the swirl flow tends to raise turbulence levels by forming a second free shear layer in the circumferential direction. Instabilities or turbulence mixing are mostly responsible for driving the near-field of a non-swirl jet, while pressure plays only a small role. However, when a tangential velocity component is superimposed on the axial one in a circular jet, both radial and axial pressure gradients are generated. These gradients could have a significant effect on changes in geometry, evolution, and interactions of the vortical structures^16^. In the literature, swirling jet flow was created using six different methods, rotating pipe, rotating honeycomb, tangential slots, tangential nozzles, deflecting vanes, and coil insert as reported in Ref.^16^.

**Figure 3:**
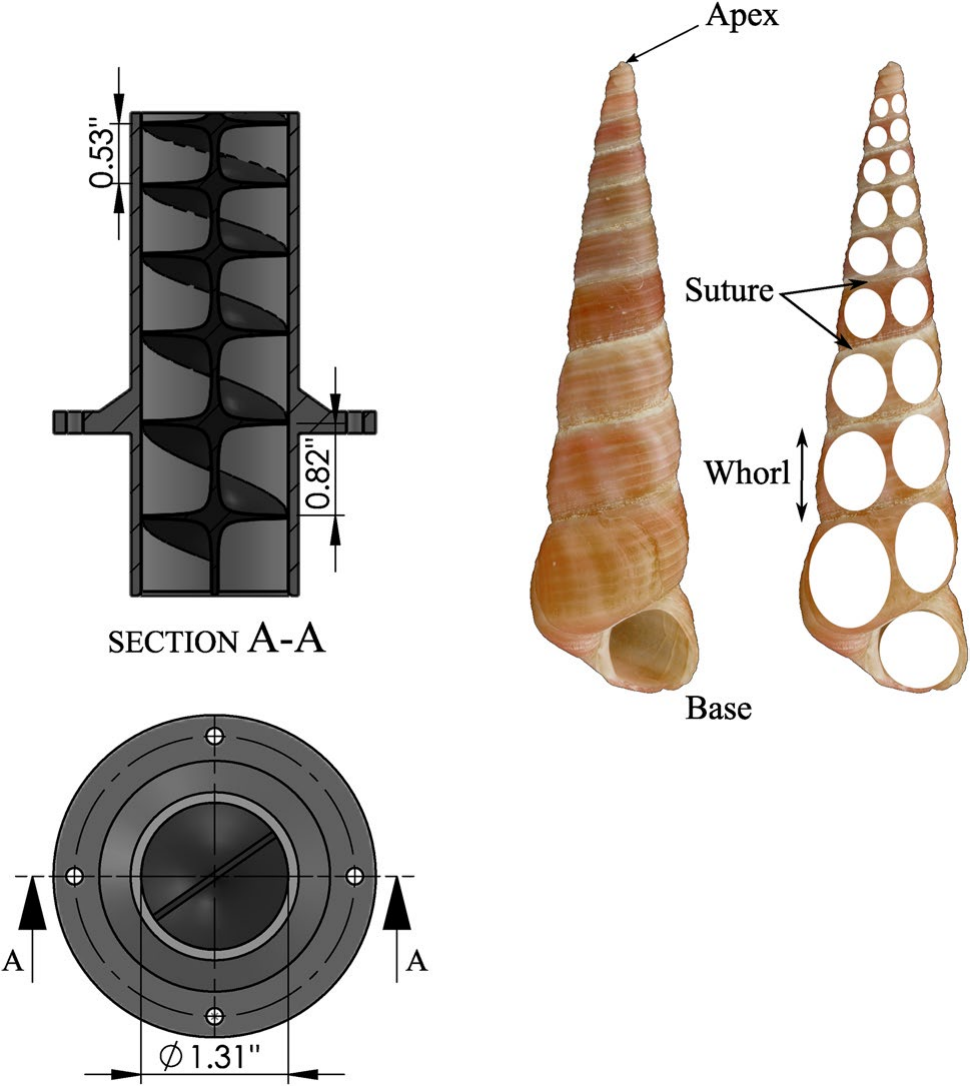
Biomimicry modeling of the snail-inspired nozzle.

In the current study, a new biomimicry swirling nozzle design inspired by a spiral snail shell structure is proposed and tested as shown in Fig. 3. A variable whorl size and spacing in the snail shells inspired us to create the snail-inspired nozzle having variable spiral pitch length along the nozzle axis as shown in Fig. 3. In order to make the design changes in LOCA holes of existing reactors easier to implement without changing reactor components, a new nozzle design should be based on a constant circular nozzle diameter. Thus, the variable spiral pitch is integrated into a circular nozzle to introduce the swirling jet flow. The acceleration of the flow inside the nozzle in this biomimetic design is not constant. It reaches a maximum value near the end of the nozzle, which increases the mixing rate of the swirling jet flow. Decreasing the spiral pitch increases the intensity of the swirling flow by increasing the number of spiral flow channels. However, the pressure drop will significantly increase due to the added resistance to flow inside the spiral channels. The main challenge in introducing a mixing feature in the existing nozzle is to keep the pressure drop similar to that in the existing nozzle. To achieve this, the pitch and size of the spiral channel should be optimized based on the pressure drop across the nozzle.

**Figure 4:**
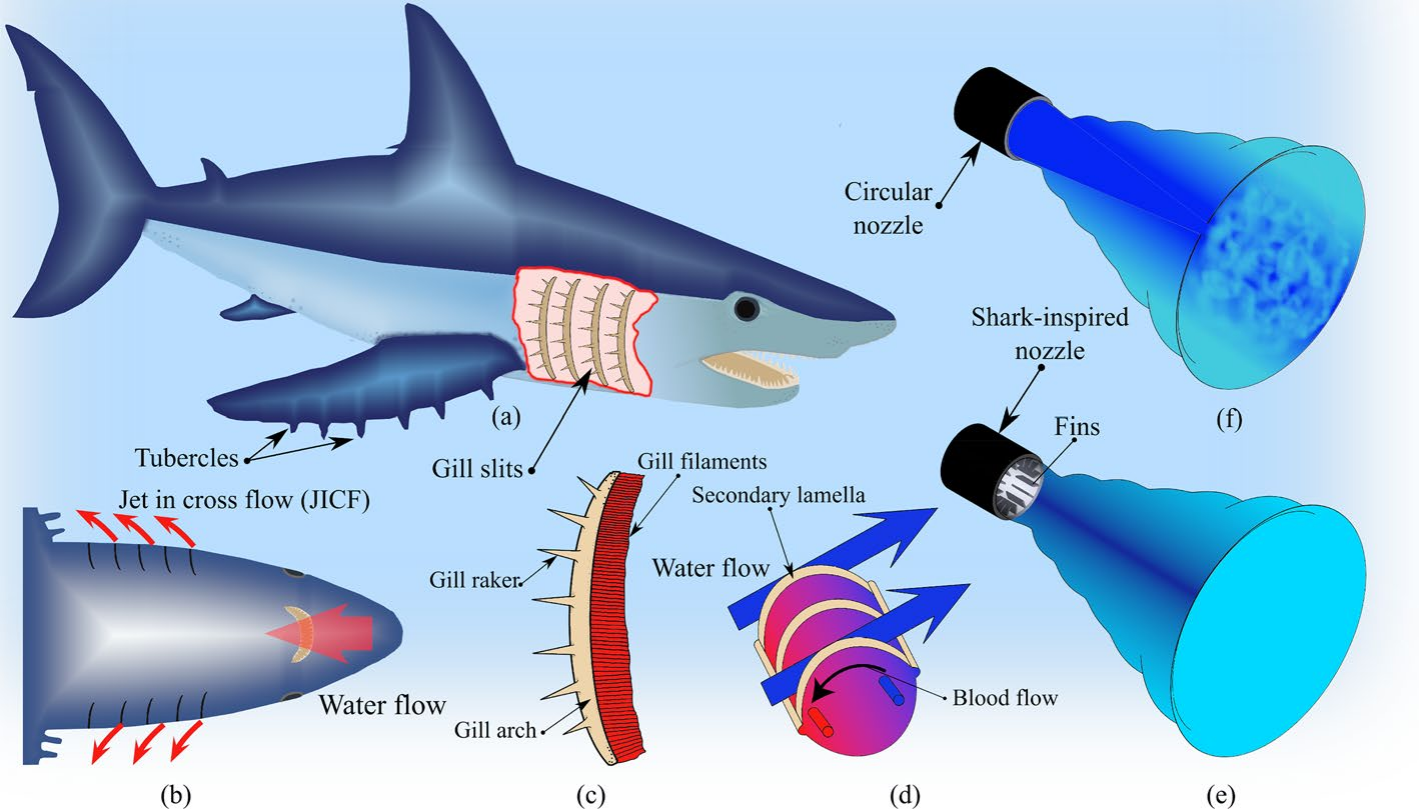
Biomimicry modeling of the shark-inspired nozzle^5^.

**Figure 5:**
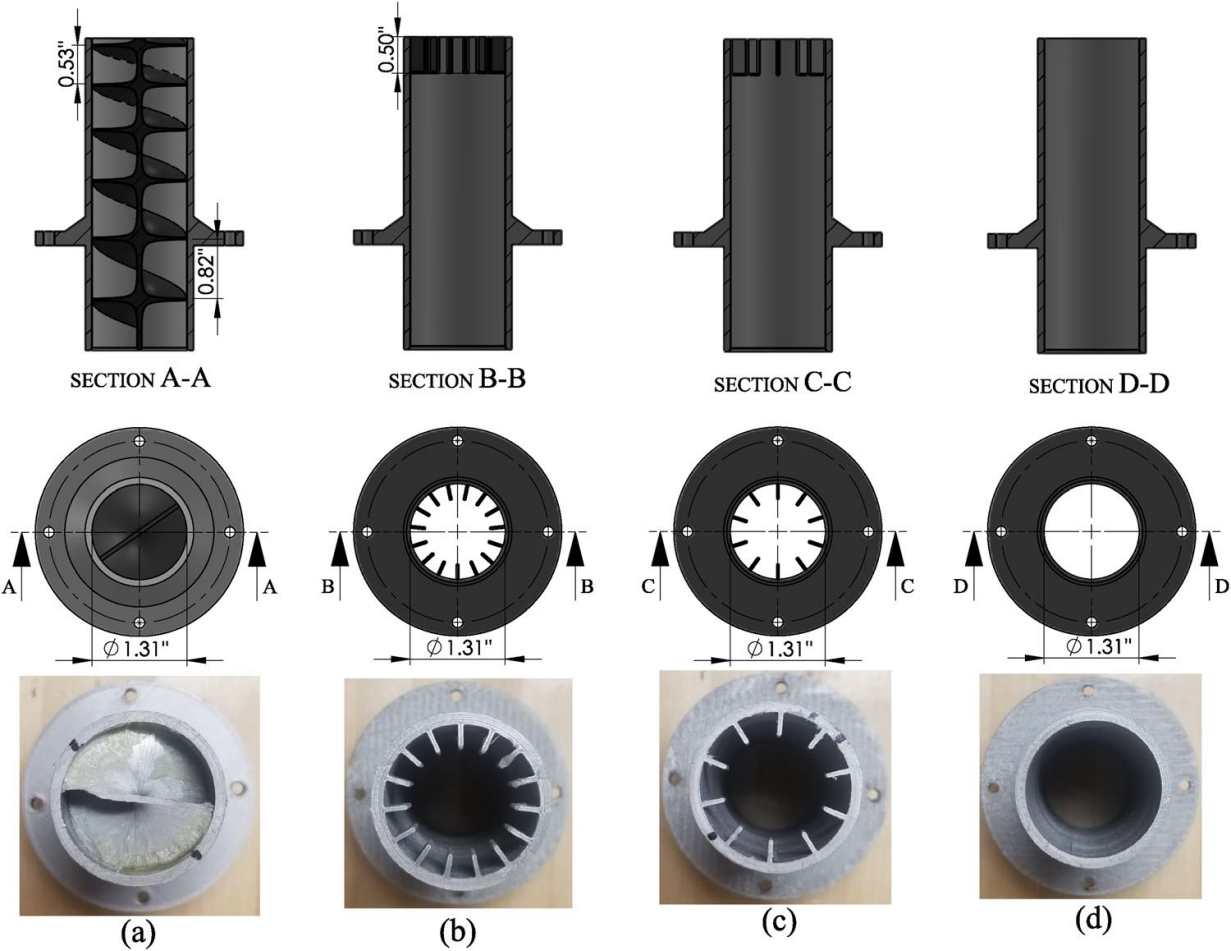
Four 3D printed nozzles: (**a**) the snail-inspired nozzle, (**b**) the shark-inspired nozzle with 15 fins, (**c**) the shark-inspired nozzle with 10 fins, and (**d**) the circular nozzle.

Gad-el-Hak and Mureithi^5^ proposed and tested a biomimetic nozzle inspired by the design of the shark gill slits to enhance the mixing of jet flow injected into a stagnant fluid. The bio-inspired fins (i.e. mechanical tabs) were attached to the circumferential base of the circular nozzle mimicking the geometry of the gill slits as shown in Fig. 4. The results showed that the shark-inspired nozzle mitigated the rod array vibrations subjected to pure jet cross-flow. However, testing the design concept of the shark-inspired nozzle for the combined jet cross-flow and axial flow-induced vibrations is not investigated in the literature. To evaluate the performance of the proposed biomimetic nozzles, a circular nozzle is first tested on fuel assembly vibrations as a reference case for the comparison between nozzles. Figure 5 shows the four nozzles that are tested in the current study to show their effects on the fuel assembly vibrations induced by the combined flow. Each nozzle has the same diameter at the nozzle base.

### Mock-up fuel rod array design

An experimental test facility is designed and built to examine the effect of the biomimetic nozzles on the fuel rod vibrations induced by the combined jet cross-flow and axial flow. PWR fuel rods are packed in a highly compact square lattice with a pitch-to-diameter ratio (*P*/*D*) of 1.32. In the Westinghouse company design^17^, the fuel rods typically form a 17x17 fuel assembly. The LOCA hole diameter is approximately covered by 3–4 rods in the fuel assembly, thus six columns facing the jet flow are selected to capture the jet flow dynamics as recommended by Gad-el-Hak et al.^6,7^. Weaver and El-Kashlan^18^ experimentally studied the effect of the number of rows on the array instability threshold under uniform flow. They recommended that the array should have at least four rows to ensure flow development to a ’steady state’ within the array. A 6x5 rod array configuration, with the six-rod side facing the jet flow, was therefore selected for testing in the lab.

**Figure 6:**
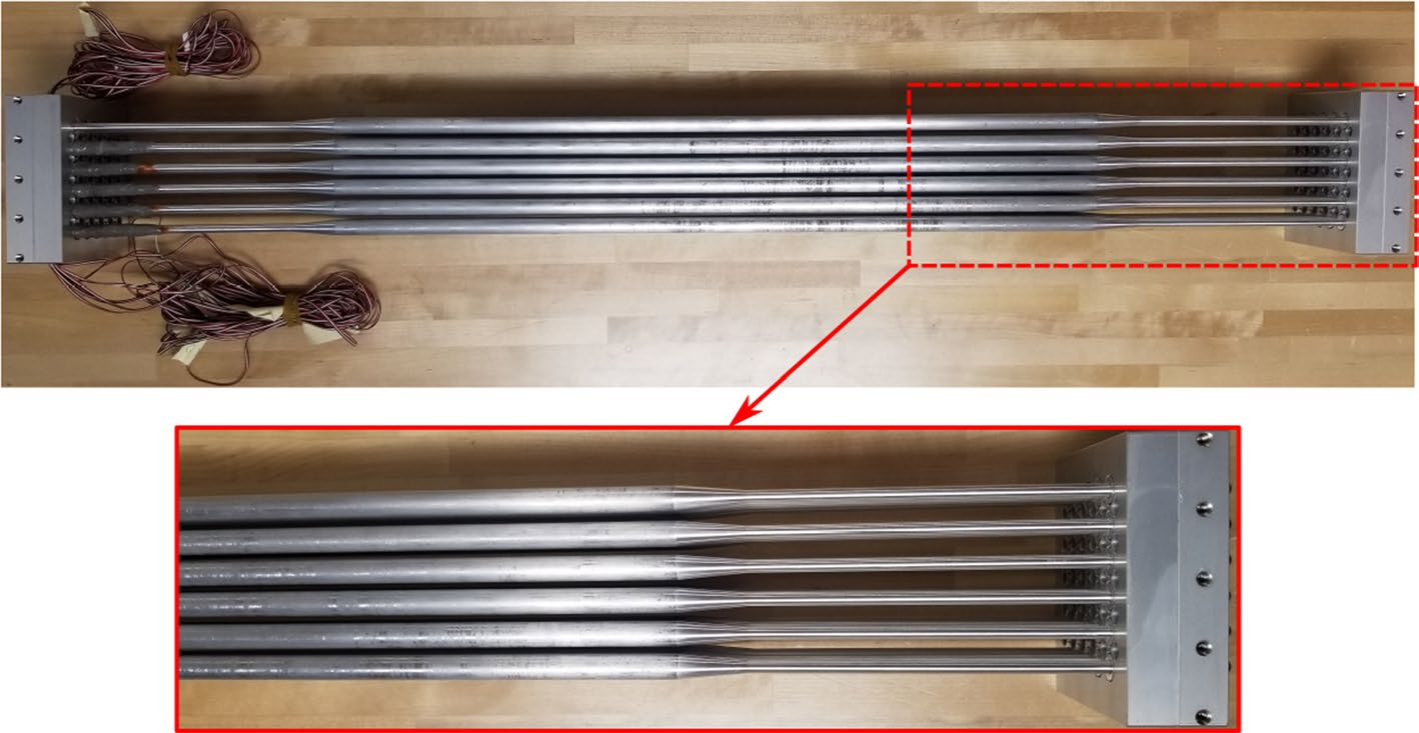
6x5 fully flexible rod bundle assembly.

The test rods, mimicking the fuel rods, are fabricated by machining solid aluminum rods to the same fuel rod diameter and rod span length. The rods are elastically mounted in the array with a specific frequency of the fuel rod. In order to achieve the rod design frequency of 35 Hz in air, the rod diameter is decreased near its end as shown in Fig. 6. The change in diameter occurs gradually with a transition angle of 5$^{\circ }$ to avoid flow separation at the entrance of the rod bundle.

**Figure 7:**
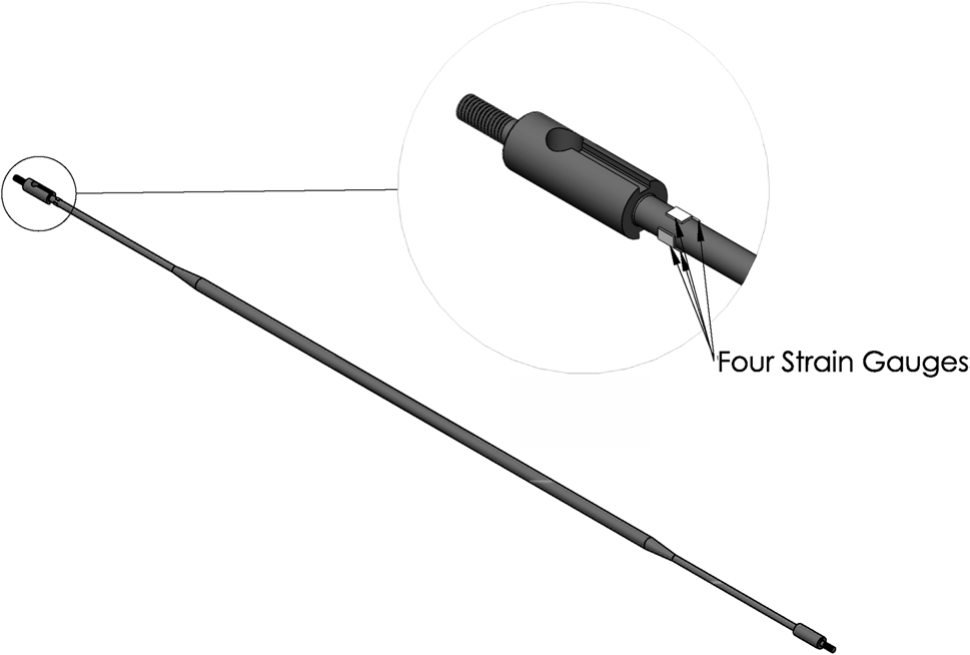
Schematic drawing showing the strain gauge installations on the flexible rod.

The main objective of this research is to measure the rod array response due to combined axial flow and jet cross-flow to determine the performance of the different nozzle types. This can be achieved by instrumenting the rods to measure the rod displacement. Instrumentation of the mock-up array is a challenge because the array is fully submerged in complex flow conditions and the array is highly compact due to the small rod and spacing dimensions. Instrumentation was successfully done by employing very small strain gauges. Four strain gauges are installed on the rod support near the clamping point as shown in Fig. 7. Each pair of gauges is connected to form a Wheatstone half-bridge circuit. The stream-wise and transverse vibrations are tracked using two half-bridge circuits installed on the rod.

**Figure 8:**
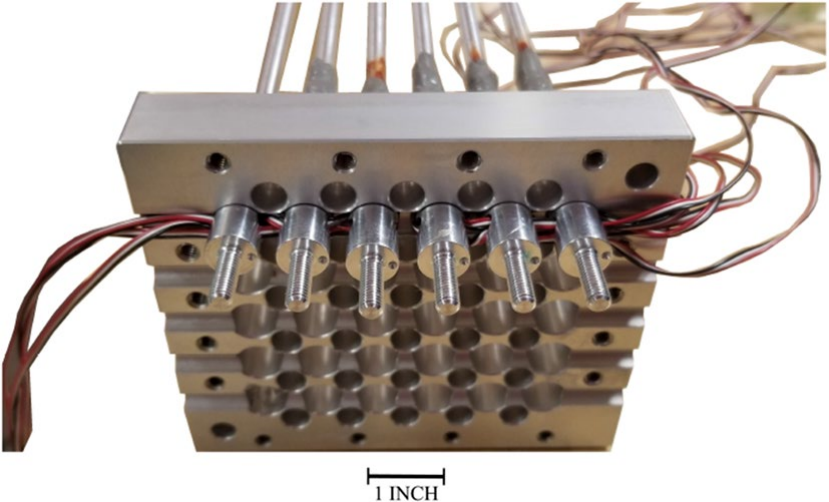
End plate design.

The rods are clamped on two plates, known as “*end plates*” as shown in Fig. 8. These plates have several functions. They serve to clamp the rods, provide openings for axial flow into the rod flow channels as well as provide a path for strain gauge wires to exit the test section. The end plates are designed to have flow holes normal to the axial flow direction and internal grooves for the strain gauge wires as shown in Fig. 8. One group of wires for the two instrumented rods is transferred from one side, and the other group is passed from the other side.

**Figure 9:**
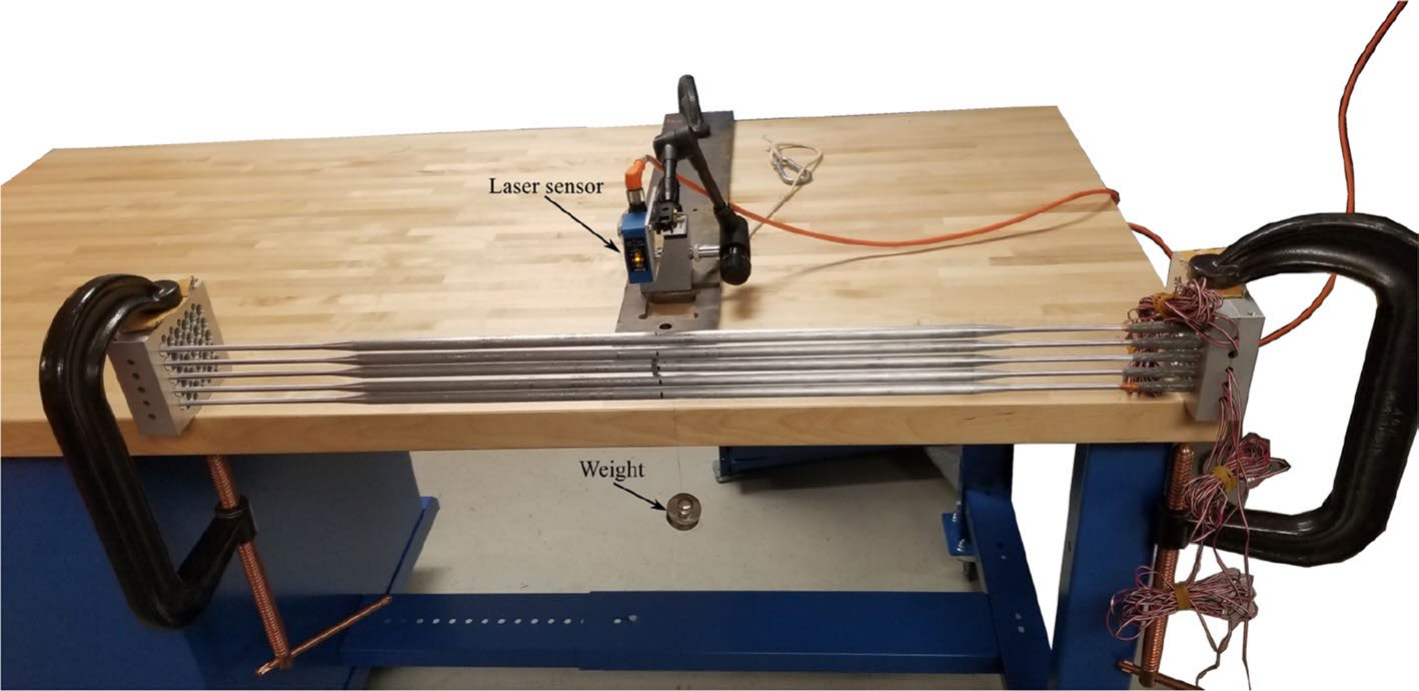
Test rig for the rod calibration.

After the strain gauge installation, each rod was tested to check the alignment of the half-bridge circuit with the rod axis. Two bridge signals were monitored while the one-rod axis was displaced to check the cross-coupling terms between the stream-wise and transverse directions. Figure 9 shows the calibration rig used to check the alignment of the strain gauges with the rod axis and to determine the calibration factor between strain and displacement. A set of calibrated weights was used to displace the mid-point of the rod with different values. The mid-point displacement was measured using a laser sensor as shown in Fig. 9. The calibration factor from strain to rod displacement is calculated based on the first-mode bending vibrations because the rod vibration occurred in the first mode.

**Figure 10:**
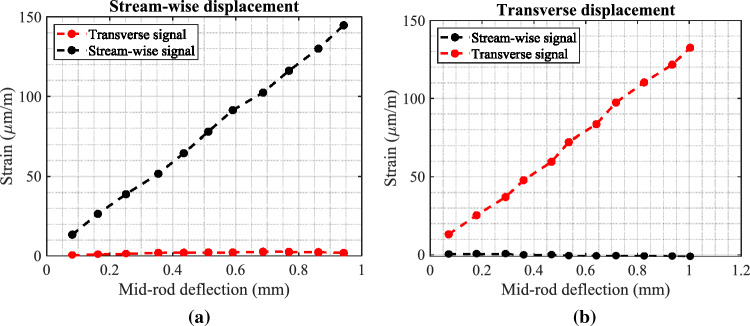
Rod calibration for the stream-wise and transverse directions: (**a**) for the stream-wise direction, and (**b**) for the transverse direction.

As shown in Fig. 10, the two signals (in the two orthogonal directions) are clearly independent of each other; hence when one direction is loaded, the other direction does not see (indicate) any deformation.

### Test section design

**Figure 11:**
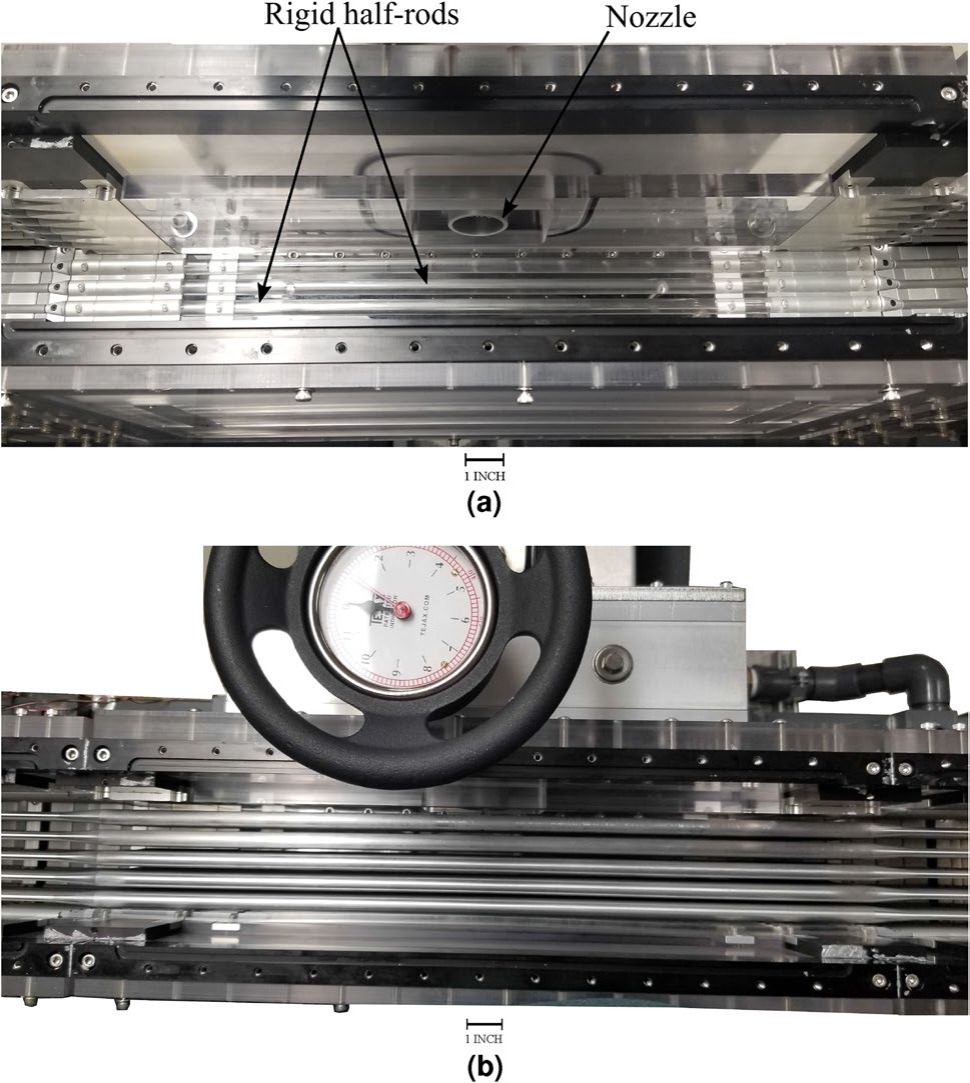
(**a**) Test section setup, and (**b**) installed the fuel rod array inside the test section.

The test section is designed to simulate the fluid dynamic conditions in the real reactor core but at room temperature. The confined flow channel around the mock-up bundle is provided by the test section side panels. These panels act as the boundary of the reduced rod array in the fuel assembly, thus one side simulating the baffle plate where the nozzle is located has a smooth surface as in the reactor. Rigid half rods are fixed on the remaining three sides to ensure flow uniformity and correct boundary conditions in the test section. Figure 11a shows part of the test section panels. The jet flow in all tests is directed toward the center of the rod array as shown in Fig. 11b.

### Test loop setup

**Figure 12:**
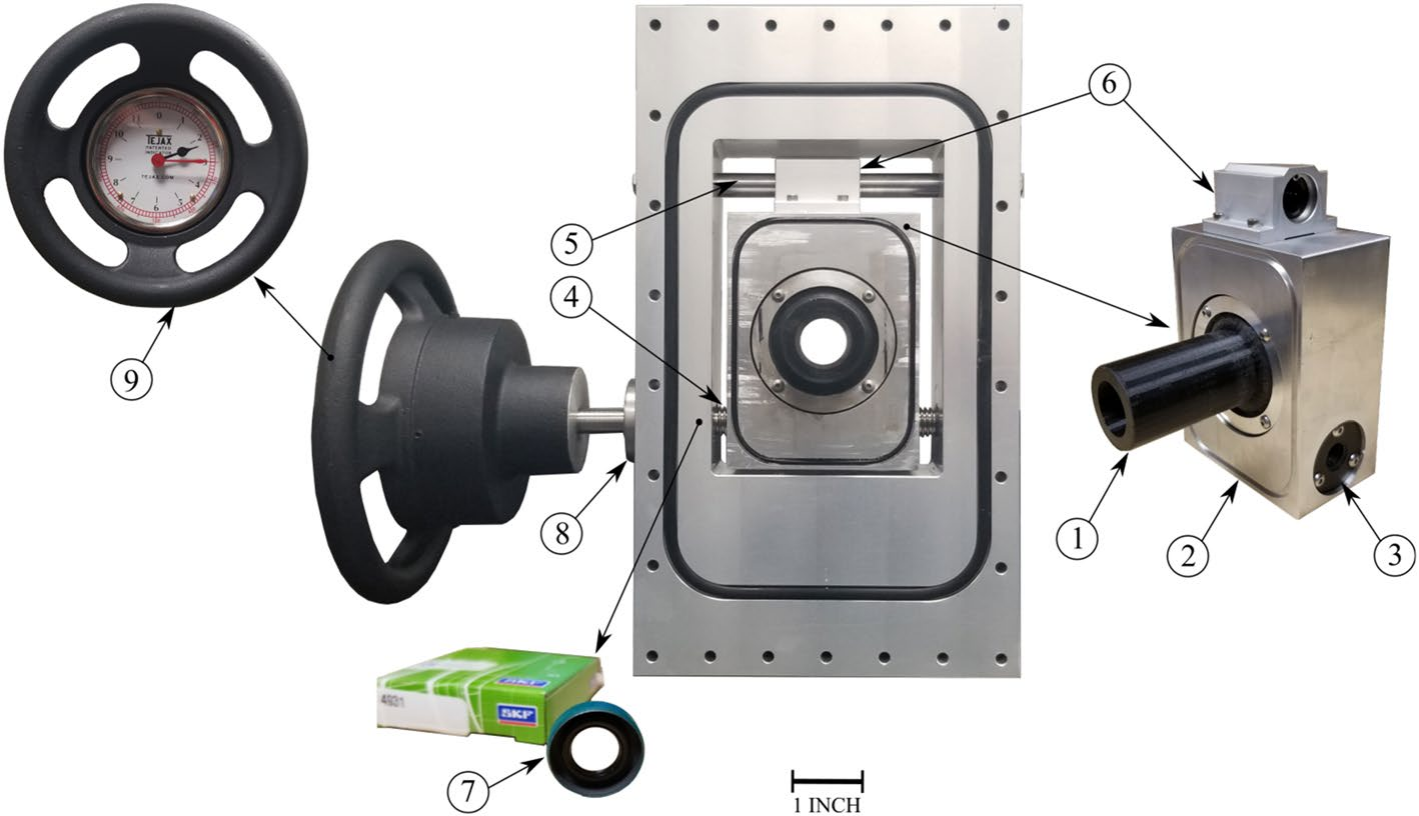
Jet flow displacement mechanism showing the main components: (1) nozzle, (2) moving plate, (3) threaded flange nut, (4) ultra-precision lead screw, (5) linear motion shaft, (6) linear ball bearing, (7) spring-loaded rotary shaft seal, (8) linear ball bearing and (9) hand wheel with the dial indicator.

**Figure 13:**
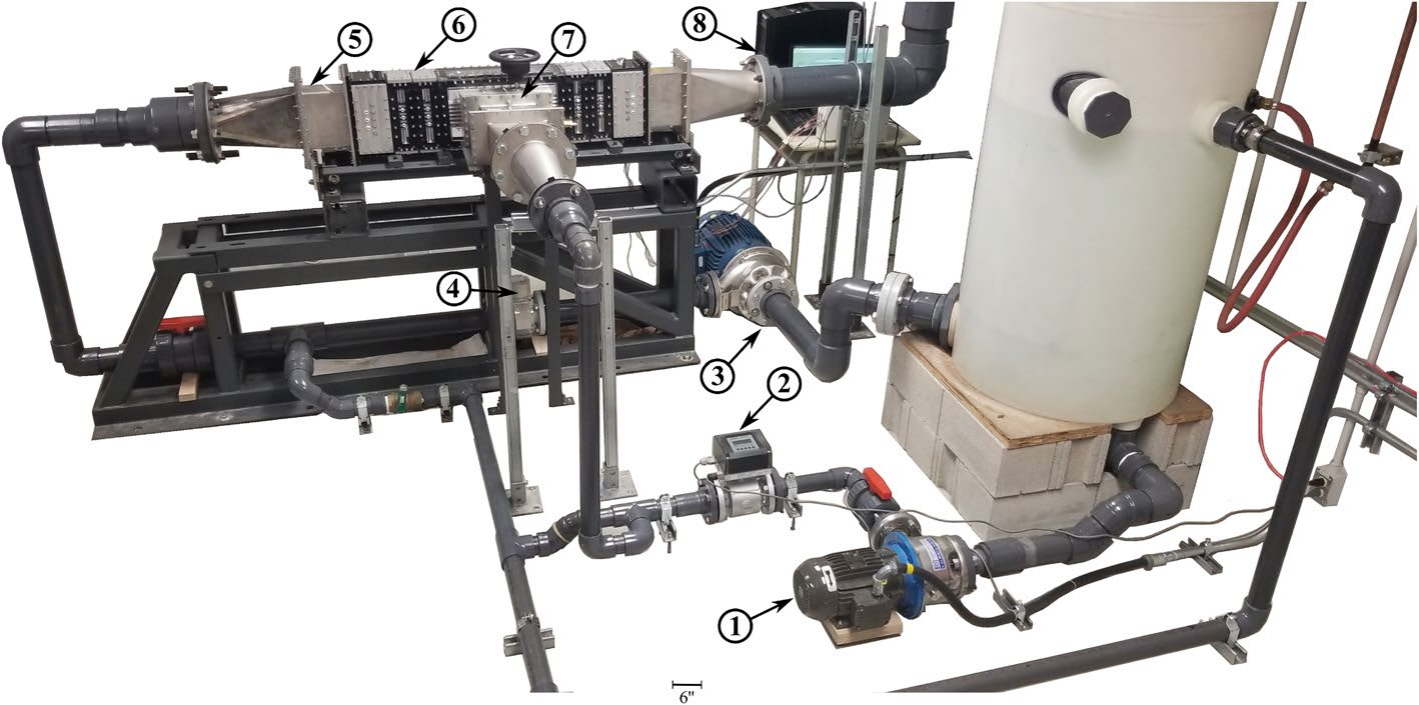
The main components of the test loop setup are labeled: (1) cross-flow pump, (2) cross-flow meter, (3) axial flow pump, (4) axial flow meter, (5) flow straightener, (6) main test section, (7) displacement jet flow mechanism, (8) data acquisition system.

Installation of pipes that bring water to the test section is the next step in completing the experimental test facility. There are two sub-loops for the combined flow: (i) the axial flow loop branch, and (ii) the jet cross-flow loop branch. Each sub-loop has a different pump capacity to provide a wide range of water flow. Pumping the axial water flow up to flow velocities in accordance with core operating conditions is performed by a 25 HP centrifugal pump. While a smaller, 2 HP, pump is used for the jet cross-flow. A specialized displacement mechanism is designed to move the jet flow (nozzle) and introduce an offset with the rod bundle centerline. As shown in Fig. 12, this mechanism consists of a 3D-printed nozzle fixed on an aluminum plate which is driven by an ultra-precision lead screw. For measuring the average flow rate in each branch, each sub-loop is connected to a flow meter. Figure 13 shows the final test loop to determine the dynamic behavior of the PWR mock-up array subjected to combined axial flow and jet cross-flow. To reduce turbulence caused by pipe connections, the flow first passes through a flow straightener (labeled 5 in Fig. 13) before it enters the rod flow channel. To reduce any extraneous vibrations, the assembled test section is fixed on a sturdy support structure as shown in Fig. 13.

## Test results

Jet in transverse flow-induced vibration is experimentally investigated for the PWR mock-up 6x5 array mimicking a reduced fuel assembly to investigate its dynamical behavior under different nozzle designs. The rod array vibration is measured by increasing the jet cross-flow rate ($Q_{Jet}$) up to the onset of an unstable vibration condition (*fluidelastic instability*) at a specific axial flow velocity. The jet flow-induced instability is studied with two different axial flow velocities $V_{Axial}$ = 1.0 and 1.5 m/s. In the following subsections, the vibratory response of the array will be presented with the three nozzles, the circular nozzle, the snail-inspired nozzle, and the shark-inspired nozzle.

### Jet flow-induced vibrations of fuel rod bundle for the circular nozzle

A total RMS of rod vibration is used in this study to obtain an equivalent response of the axisymmetric flexible rod in both directions. The RMS response is normalized by the inter-rod gap (G = $P-D$). It is defined as follows:

1 $$\begin{aligned} {\rm{RMS}} =\sqrt{{\rm {RMS}}_{x}^{2}+{\rm{RMS}}_{y}^{2}}; \;\; {\rm RMS} (\% {\rm G})=\frac{ {\rm RMS}}{\rm {G}}\times 100, \end{aligned}$$

where RMS$_{x}$ and RMS$_{y}$ are the root mean squares of the stream-wise response and the transverse response relative to the jet cross-flow, respectively.

**Figure 14:**
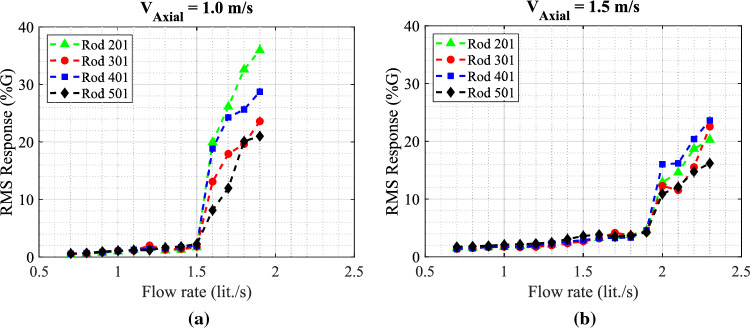
RMS response of the four rods in the fully flexible array obtained with the circular nozzle for the two tested axial flow velocities: (**a**) $V_{Axial}$ = 1.0 m/s and (**b**) $V_{Axial}$ = 1.5 m/s.

**Figure 15:**
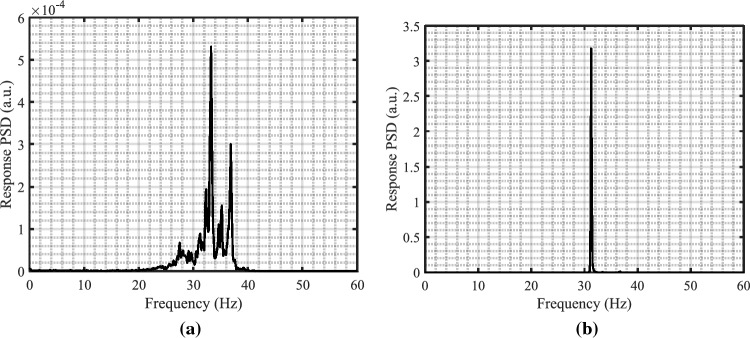
PSD plots in the transverse direction of rod 401 with the two jet flow rates: (**a**) $Q_{Jet}$ = 1.1 lit./s and (**b**) $Q_{Jet}$ = 1.8 lit./s.

The four instrumented rods are placed in the middle of the first row facing the jet flow. Figure 14 illustrates the behavior of the rod bundle subjected to the circular jet in transverse flow for two tested axial flow velocities $V_{Axial}$ = 1.0, and 1.5 m/s. The RMS vibration response of the four rods is obtained by increasing the flow rate through the nozzle ($Q_{Jet}$) up to the critical condition of the array stability. The dynamical behavior of the mock-up array can be divided into two regions based on the jet flow rate: region (I) $Q_{Jet}$ from 0.7 to 1.5 lit./s and region (II) $Q_{Jet}$ from 1.5 to 1.9 lit./s. In region (I), the turbulence in the combined flow induced small vibration amplitudes, less than 2%G, in the array. This is confirmed by the wide band power spectral density (PSD) plot as shown in Fig. 15a. The jet flow rate $Q_{Jet}$ = 1.5 lit./s marks the stability threshold for the rod bundle. Above this limit, the response increases sharply reaching 38%G at the maximum jet flow rate tested. This vibration behavior corresponds to the phenomenon of fluidelastic instability (FEI), where the rod vibrates sinusoidally at its natural frequency as shown in Fig. 15b.

Increasing the axial flow velocity increases the array stability limit as shown in Fig. 14b. The critical jet flow rate is increased from 1.5 lit/s to 1.9 lit./s. In addition, the maximum vibration amplitude in the array is relatively lower than that in the $V_{Axial}$ = 1.0 m/s case. This stabilizing effect of axial flow could be attributed to two parameters. Firstly, the axial flow induced damping in arrays as reported in previous work^2^. Secondly, the injected jet flow is deflected by the axial flow which means that increasing the axial flow adds more resistance to the jet flow which impedes jet penetration deeper into the array.

### Jet flow-induced vibrations of fuel rod bundle for the snail-inspired nozzle

**Figure 16:**
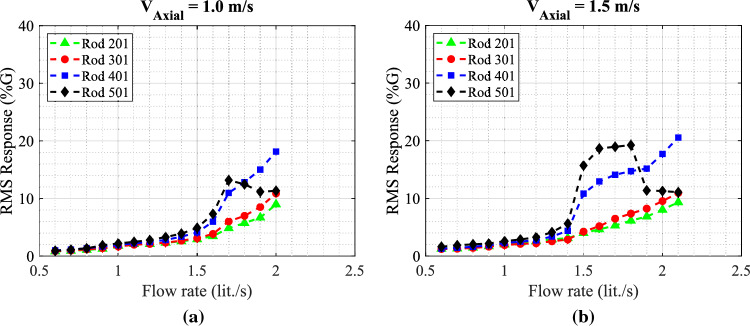
RMS response of the four rods in the fully flexible array obtained with the snail-inspired nozzle for the two tested axial flow velocities: (**a**) $V_{Axial}$ = 1.0 m/s and (**b**) $V_{Axial}$ = 1.5 m/s.

The collected experimental data for the rod array vibrations obtained with the circular nozzle provide a reference case for evaluating the performance of the snail-inspired nozzle. The dynamical behavior of the mock-up array is obtained by increasing the jet flow rate injected through the biomimetic nozzle in the same range as the circular nozzle case. Figure 16 shows the rod array vibration results for the snail-inspired nozzle. The variable pitch spiral structure in the nozzle demonstrates its impact in reducing the large amplitude vibration by the swirling effect. The amplitude is reduced by 50% on switching from the circular nozzle to the snail-inspired nozzle for the $V_{Axial}$ = 1.0 m/s case. In addition, a reduction of 35% in the maximum vibration amplitude is obtained at the higher axial flow case, $V_{Axial}$ = 1.5 m/s. For $V_{Axial}$ = 1.5 m/s, rod 501 behaves differently than the other rods. Its response goes up to 20%G, then it decreases to 10%G with increasing the jet flow rate. This phenomenon is attributed to synchronization (lock-in) between the flow periodicities and the structural response, which could cause strong resonance oscillations. Increasing the flow rate eliminates the coupling between the structure and the flow periodicities. The results of the snail-inspired nozzle show an interesting effect of suppressing the jet in transverse flow-induced vibrations by creating a swirl flow.

### Jet flow-induced vibrations of fuel rod bundle for the shark-inspired nozzle

**Figure 17:**
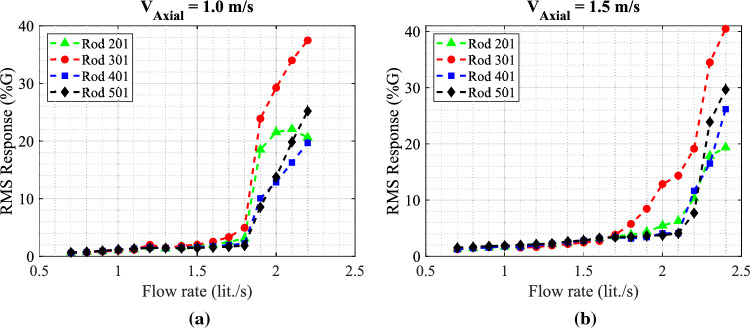
RMS response of the four rods in the fully flexible array obtained with the 10-fin shark-inspired nozzle for the two tested axial flow velocities: (**a**) $V_{Axial}$ = 1.0 m/s and (**b**) $V_{Axial}$ = 1.5 m/s.

The shark-inspired nozzles (having 10 fins & 15 fins) were tested to investigate the performance of the bio-inspired mechanical tabs integrated into the nozzle on mitigating the fuel assembly vibration. The shark-inspired with 10 fins was first tested, then the fin number was increased to 15 fins to show its effect on the array stability condition. Figure 17 shows the RMS response of the four rods obtained with the 10 fins bio-inspired nozzle and two axial flow velocities $V_{Axial}$ = 1.0 and 1.5 m/s cases. As seen in Fig. 17, the bio-inspired mechanical tabs significantly impact the fluidelastic behavior of the rod array. The critical limit of the jet flow rate at which instability initiated is delayed, from 1.5 lit./s to 1.8 lit./s (+20%) for $V_{Axial}$ = 1.0 m/s case by using the 10-fin shark-inspired nozzle instead of the circular nozzle. Furthermore, the maximum vibration amplitude obtained with the circular nozzle is damped by 87.5% at the stability limit. Unlike the snail-inspired nozzle results, the rate of increase of the vibration response above the stability threshold is sharp, which is similar to the circular nozzle case. The vibration mitigation by the shark-inspired nozzle still exists at the higher axial flow velocity case, $V_{Axial}$ = 1.5 m/s. The stability threshold is elevated by 10.5%. Compared with the results obtained with the circular nozzle at $V_{Axial}$ = 1.0 m/s, the vibrations of the three rods 201, 401, and 501 are suppressed by 66.7%, while the vibration of rod 301 is almost the same for both nozzles. For $V_{Axial}$ = 1.5 m/s case, the reduction percentages of the rod responses for rod 401, 501, and 201 are 75%, 66.7%, and 57%, respectively. The finding that biomimetic nozzle designs have an important impact on mixing rate is derived from the comparison of results obtained with three types of nozzles. These nozzles would lead to a major decrease in vibration in the rod bundle.

**Figure 18:**
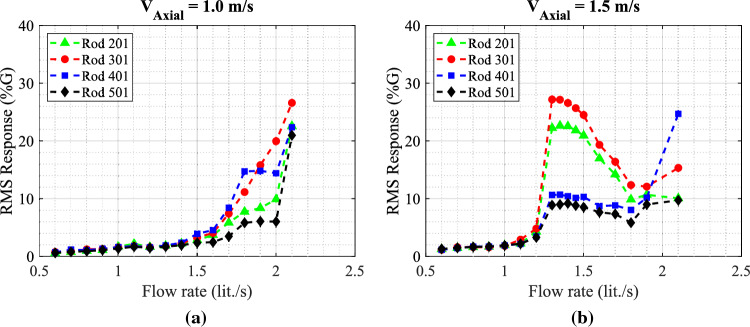
RMS response of the four rods in the fully flexible array obtained with the 15-fin shark-inspired nozzle for the two tested axial flow velocities: (**a**) $V_{Axial}$ = 1.0 m/s and (**b**) $V_{Axial}$ = 1.5 m/s.

When assessing the mixing rate and water flow resistance through the shark-inspired nozzle, the number of fins is an essential parameter. The fin number should carefully be selected because increasing the number of fins is linked to higher pressure drops. In order to investigate how the fin number affects the rod array vibration, we have tried a different shark-inspired nozzle with 15 fins. Figure 18 shows the RMS response of the four rods obtained with the 15 fins shark-inspired nozzle and two axial flow velocities $V_{Axial}$ = 1.0 and 1.5 m/s cases. In the case of $V_{Axial}$ = 1.0 m/s, increasing the number of fins from 10 to 15 reduces the maximum vibration amplitude obtained with the 10-fin shark-inspired nozzle by 23.5%. In comparison with the circular nozzle results, vibration amplitude reduction for the four rods is 74% for rod 201, 70% for rod 501, 53% for rod 401, and 41% for rod 301. In addition, the critical flow rate is delayed by 33.3%. The 15-fin shark-inspired nozzle shows a greater effect compared to the 10-fin nozzle on suppressing the vibration caused by the jet in transverse flow for $V_{Axial}$ = 1.0 m/s. However, the fluidelastic behavior of the mock-up array is different as shown in Fig. 18b for the higher axial flow velocity case, $V_{Axial}$ = 1.5 m/s. The two rods located in the upper half of the array behave completely differently than the other two rods. Above $Q_{Jet}$ = 1.2 lit./s, the responses of two rods 201 and 301 increase from 5%G to 27%G and to 22%G, respectively. Then, by increasing the jet flow rate, the responses reduce again to reach 10%G at $Q_{Jet}$ = 1.8 lit./s. While two rods 401 and 501 have a stabilization behavior with increasing the jet flow rate. Above $Q_{Jet}$ = 1.2 lit./s, the responses of these two rods increase from 5%G to 10%G, then they are almost constant with increasing the jet flow rate. To conclude, the bio-inspired nozzles prove their ability to reduce the fuel assembly vibrations for the complex flow condition of jet-in-transverse flow. The snail-inspired nozzle shows its advantage in switching from a sharp increase in amplitude to a gradual increase with increasing the jet flow rate. The advantages of using shark-inspired nozzles include a dynamic response quite similar to a circular nozzle while the stability threshold is elevated or delayed.

## Discussion

**Figure 19:**
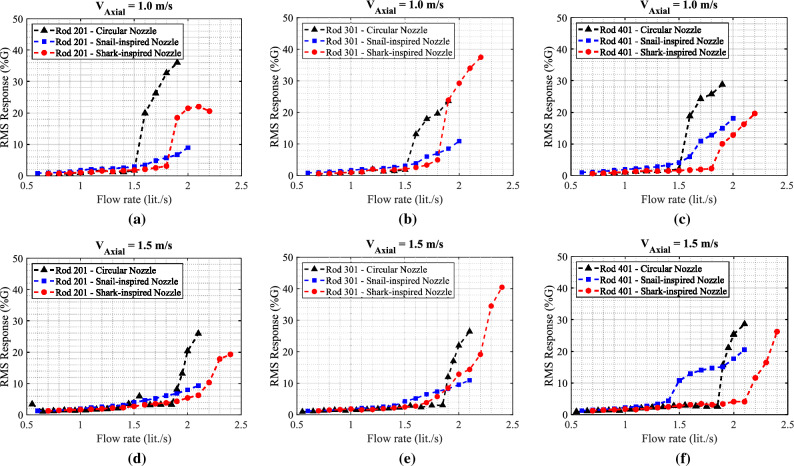
Comparison of RMS response of rods 201, 301 and 401 obtained with the two tested axial flow velocities $V_{Axial}$ = 1.0 m/s and 1.5 m/s with the three different nozzles: (**a**) rod 201 & $V_{Axial}$ = 1.0 m/s, (**b**) rod 301 & $V_{Axial}$ = 1.0 m/s, (**c**) rod 401 & $V_{Axial}$ = 1.0 m/s, (**d**) rod 201 & $V_{Axial}$ = 1.5 m/s, (**e**) rod 301 & $V_{Axial}$ = 1.5 m/s, and (**f**) rod 401 & $V_{Axial}$ = 1.5 m/s.

The fundamental problem addressed in this paper is the following: for safety reasons, reactor fuel rods are subjected to a high-speed jet flow. Unfortunately, the same jet flow can destabilize the rods, which can result in instability, vibration, and mechanical failure. The work reported here proposes a solution that eliminates the instability problem by modifying the jet by using biomimetically designed nozzles. The new nozzles are therefore better because the designs eliminate the vibration problem. The physical process underlying the stabilization is the efficient flow mixing and flow momentum (axial) reduction that is achieved by the new designs. Two biomimetic nozzles, the snail-inspired nozzle, and the shark-inspired nozzle, are proposed and tested to mitigate the jet in transverse flow-induced vibrations by providing efficient and rapid mixing between the jet flow and axial flow. The performance of the bio-inspired nozzles is evaluated versus the circular nozzle-induced rod bundle vibrations. The results demonstrate the potential advantages of using nozzles invented by nature to mitigate the vibration rod arrays subjected to combined jet cross-flow and axial flow as shown in Fig. 19. Bio-inspired tabs from sharks are used to improve the mixing effect of circular jet flow by introducing stream-wise vortices to increase the dissipation of jet flow into the axial flow as demonstrated in Ref.^19^. The delaying effect of the shark-inspired nozzle on the stability limit at $V_{Axial}$ = 1.5 m/s (10.5%) is relatively less than that occurred at $V_{Axial}$ = 1.0 m/s (20%). This could be due to an increase in the axial flow velocity intensifying the turbulence intensity in the array with turbulence coming from the shark-inspired nozzle, which would increase the response caused by turbulence-induced vibration. Furthermore, the snail-inspired nozzle produces a swirling jet flow which forms a second free shear layer in the circumferential direction. This could have a significant effect on the vortical structures of jet flows. More importantly, the impact of changing the nozzle shape from a circular design on the pressure drop across the nozzles needs to be determined.

**Figure 20:**
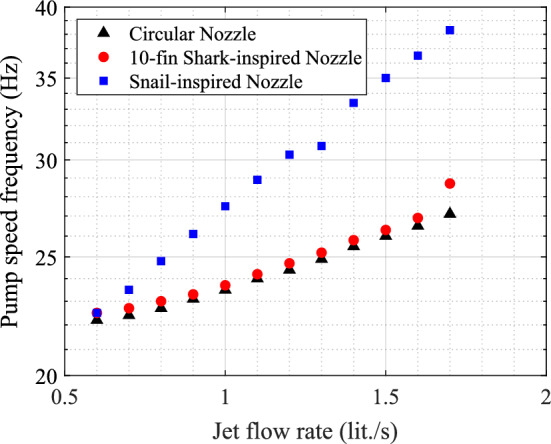
Comparison of pump speed frequencies obtained with the three nozzles, the circular nozzle, the 10-fin shark-inspired nozzle, and the snail-inspired nozzle.

The pressure drop across the nozzle is correlated to the frequency of the pump motor speed. Therefore, the pump speed frequency is recorded while the jet flow rate is increased in the three nozzles, the circular nozzle, the 10-fin shark-inspired nozzle, and the snail-inspired nozzle as shown in Fig. 20. The required pumping power for the jet flow through the shark-inspired nozzle is slightly increased. The increase in the pump speed frequency caused by attaching very thin fins to the circular nozzle, is, however, less than 0.5 Hz. For the two nozzles, the circular nozzle and the 10-fin shark inspired, the trend of the pump speed with the flow rate is similar as shown in Fig. 20. However, the snail-inspired nozzle design adds more flow resistance, and a significant increase in required pumping power for this nozzle is observed when looking at the frequency-flow rate relation. Two key parameters could be behind this, an increase in the nozzle surface area in contact with the flow and a change in flow characteristics within the nozzle (i.e. rotational flows). The surface area of the snail-inspired nozzle is 114% larger relative to the circular nozzle surface area; however, the (surface area) increase for the 10-fin shark-inspired nozzle is 10%. The main advantage of using the shark-inspired nozzle is keeping the same pumping capacity that is provided for the circular nozzle. On the other hand, the snail-inspired nozzle has an advantage in limiting maximum vibration amplitudes, for slightly more pumping power.

## Conclusions

This research addresses a biomimetic solution to the problem of nuclear fuel rod fretting and wear in some pressurized water reactors (PWRs). PWR fuel rods are subjected to combined axial flow and jet cross-flow at certain locations along the fuel rod span. The combined flow has been observed to induce vibrations in fuel assemblies, resulting in dynamic interaction between fuel rods and their supports (i.e. spacer grids). Leakage from fuel rods could result from this dynamic interaction. The ultimate solution to mitigate the vibration of fuel rods is to enhance the mixing between the jet flow and the axial coolant flow inside the reactor core.

Marine biomimetics is used to propose new designs based on circular nozzle shapes. The variable whorl size and spacing in snail shells were a source of inspiration to create a swirling jet flow to increase the mixing features of jet flow. In this design, the dynamics of jet flow are converted from pure linear momentum dominated into angular momentum dominated by switching from the straight wall circular nozzle to the snail-inspired nozzle. In a second design, biomimetic tabs inspired by gill rakers in sharks are incorporated into the circular nozzle to investigate their effects on reducing vibrations of fuel rods caused by combined axial flow and jet cross-flow.

A PWR mock-up bundle is designed, fabricated, and instrumented to investigate the effects of the three nozzle designs, the circular nozzle, the snail-inspired nozzle, and the shark-inspired nozzle on the rod array vibration. The ability of biomimetic nozzles to reduce the rod array response is demonstrated in the tests. The experimental results show that:

(i) For the circular nozzle, the combined axial flow and jet cross-flow induced instability in the mock-up array. The critical jet flow velocity depends on the axial flow velocity parallel to the fuel rods. Increasing $V_{Axial}$ from 1.0 m/s to 1.5 m/s elevates the stability threshold by 27%.
(ii) The snail-inspired nozzle has a significant effect by mitigating the vibration amplitude of the combined flow-induced vibrations. For the $V_{Axial}$ = 1.0 m/s case, the amplitude is suppressed by 50% by switching from the circular nozzle to the snail-inspired nozzle; while 35% reduction in the maximum vibration amplitude is obtained for $V_{Axial}$ = 1.5 m/s. The instability delay effect of this biomimetic nozzle is attributed to the swirling effect of jet flow.
(iii) The shark-inspired nozzle shows its potential impact by mitigating the vibration amplitude and delaying the stability threshold of the mock-up array under the combined flow. The critical jet flow velocity is delayed by 20% and the vibration amplitude is mitigated by 87.5% in comparison with the circular nozzle results for the case of $V_{Axial}$ = 1.0 m/s.
(iv) The pressure drop across the new biomimetic nozzle designs is determined as a function of the pump speed. In the shark-inspired nozzle, the required pumping capacity is quite similar to that of circular nozzles which gives a great benefit for replacing circular nozzles with shark-inspired nozzles without changing pump capacity.
(v) Due to the spiral surface in the snail-inspired nozzle, the water flow faces larger friction resistances in addition to adding resistances to rotate the flow. Thus, a higher pump speed (power) is needed to provide the same flow rate as in the shark-inspired nozzle. On the other hand, the rod array response obtained with the snail-inspired nozzle is less violent than those obtained with the circular nozzle and the shark-inspired nozzle.

For a wide range of applications, highly effective mixing nozzles are very desirable. The proposed marine biomimetic nozzles, based on a circular shape, can be (easily) integrated into the circular nozzles to improve their mixing efficiencies. Further experiments and studies are needed to extend the use of biomimetic nozzles in a wide range of applications requiring rapid and effective mixing, such as chemical reactors, fuel injection systems, or combustion chambers. In addition, visualization of jet flow from the bio-inspired nozzles will be done in order to address the main flow features for the biomimetic nozzles.

## Data Availability

The datasets generated and/or analyzed during the current study are not publicly available due to the inclusion of proprietary information on industrial applications (in nuclear engineering) but are available from the corresponding author upon reasonable request.
